# Effects of Virtual Reality Pilates Training on Duration of Posture Maintenance and Flow in Young, Healthy Individuals: Randomized Crossover Trial

**DOI:** 10.2196/49080

**Published:** 2023-10-19

**Authors:** Sung Je Park, Jea Woog Lee

**Affiliations:** 1 College of Sport Chung-Ang University Anseong-si Republic of Korea; 2 Intelligence Information Processing Lab Chung-Ang University Seoul Republic of Korea

**Keywords:** virtual reality, Pilates, exercise program, flow, duration of posture maintenance, sport video data analytics, video, data analytics, sport, sports, exercise, physical activity, posture, VR, balance, movement, self-reported, patient reported

## Abstract

**Background:**

This study explored the use of virtual reality (VR) technology to enhance the effectiveness and duration of low-intensity movements and postures in Pilates-derived exercises. We postulate that by leveraging the flow state in VR, individuals can engage in these exercises for longer periods while maintaining a high level of flow.

**Objective:**

The purpose of this study was to compare differences in posture maintenance and flow between VR Pilates training and conventional Pilates training, and the correlation between the 2 factors.

**Methods:**

The 18 participants in each group received either VR training or conventional training and were switched to the other training type after a 2-day wash-out period. Each group performed Pilates movements in a VR environment and a conventional environment, divided into 4 types. After training sessions, participants were evaluated for flow using a self-report questionnaire. In addition, a sports video analysis program was used to measure the duration of posture maintenance in 2 video-recorded sessions. Repeated-measures ANOVA and correlation analysis were performed on the measured duration of posture maintenance and flow scores. In all cases, the statistical significance level was set at *P*<.05.

**Results:**

Results for the duration of posture maintenance verification by type showed that simple behavior (*F*_1,16_=17.631; *P*<.001), upper body–arm coordination behavior (*F*_1,16_=6.083; *P*=.04), upper body–leg coordination behavior (*F*_1,16_=8.359; *P*<.001), and whole-body coordination behavior (*F*_1,16_=8.426; *P*<.001) all showed an interaction effect at *P*<.05. Flow (*F*_1,16_=15.250; *P*<.001) also showed an interaction effect. In addition, significant correlations were determined between duration of all types of posture maintenance and flow in the VR training group at *P*<.05.

**Conclusions:**

Our results indicate that VR Pilates training may be more useful than conventional Pilates training in improving the duration of posture maintenance and that it promotes a significantly higher degree of flow when compared with conventional Pilates training.

## Introduction

Virtual reality (VR) technology provides a virtual environment using virtually created graphics or images [1]. VR was previously seen as encompassing the implementation of virtual graphics—including 2D or 3D graphics—to simulate the real world, irrespective of the type of VR device used [2]. Currently, however, VR has come to be defined as a technology that induces users to enter a virtual world while wearing a head-mounted display (HMD) that completely blocks the wearer’s visual senses [3]. This flow eventually digitizes the positions and directions of the real world (and those who live in it) while providing the wearer with 3D images, thereby affecting their movements [4].

Previous studies have emphasized the potential benefits of VR technology in certain specialty areas—such as education, training, and entertainment—with its usefulness further expanded to other disciplines [5]. The application of VR technology in sports is an innovative development for overcoming barriers through effective training methods [6,7]. This justifies the great academic interest in the applicability of VR technology to sports. Specifically, VR-based fitness effectively promotes muscle growth and aerobic capacity [8]. Moreover, VR technology is effective at improving physical activity in sports and military training [9]. A VR-based exercise rehabilitation program was effective in improving the physical strength of patients undergoing hemodialysis and in improving psychological outcomes—such as quality of life, depression, and anxiety—in those with cardiovascular diseases [10-12].

Previous studies have examined the effects of sports participation in VR environments on motivation and enjoyment. This was followed by studies showing that a high degree of flow is closely associated with VR sports games. Thus, these studies have demonstrated the effects of psychological factors [13,14]. Furthermore, mind-body physical activities—such as yoga or Pilates—conducted in a VR environment can simulate real situations [15].

Flow is defined as the degree to which individuals concentrate on an object in response to specific stimuli and can be expressed as a holistic sensation that individuals perceive while acting holistically. Moreover, flow is an inherently valuable concept closely associated with motivation, as it induces individuals to be fully engaged in an activity, experience it as intrinsically rewarding, and pursue it even without achieving an ultimate goal [16,17]. A recent study highlighted the impact of flow in the context of physical exercise; it showed that flow is closely associated with intention for sustainable use of Wii Fit or smartphone-based fitness apps [17].

Previous studies have shown that VR technology is effective in creating a state of flow because of its flow sensory and physical affordances [13,18,19]. Along the continuum of these previously published studies, we hypothesize that flow might be involved in the duration of posture maintenance in the context of VR Pilates. Therefore, we have conducted this study to assess the efficacy of VR Pilates in improving the duration of posture maintenance and its ability to promote a significantly higher degree of flow compared to traditional Pilates among typical healthy adults.

## Methods

### Study Design and Participants

Recruitment was conducted through an advertisement posted on the internet board of the College of Sport of Chung-Ang University. Participants were instructed to send a message of intent to participate to the researcher’s messenger address posted in the advertisement. The recruitment period was from February 2021 to April 2021. Participants were included if they were male Korean nationals aged ≥20 years attending university with a major in physical education; participants’ physical health, along with confirmation of an absence of mental illness, was confirmed by a health check-up program at a university-affiliated hospital. Participants were excluded if they had a history of head trauma; abused substances such as alcohol, tobacco, or drugs; were unable to understand the study and consent procedures; or had a confirmed diagnosis of a mental or psychiatric disorder [20]. For this study, we estimated the sample size using G* Power (Heinrich-Heine-Universität). To do this, we performed a repeated-measures ANOVA and used a type I error of .05, an effect size of 0.25, a statistical power of 0.9, and a drop-out rate of 10%; we also conservatively set the correlation between the repeated measures at 0. Therefore, we estimated the sample size to be a minimum of 18, and we enrolled 18 participants in this prospective, randomized, crossover trial. All 18 participants completed the trial; their age, height, and weight were measured as 24.17 (SD 2.81) years, 177.06 (SD 3.65) cm, and 75.21 (SD 9.49) kg, respectively. In this study, no participants presented with symptoms of motion sickness.

**Figure 1:**
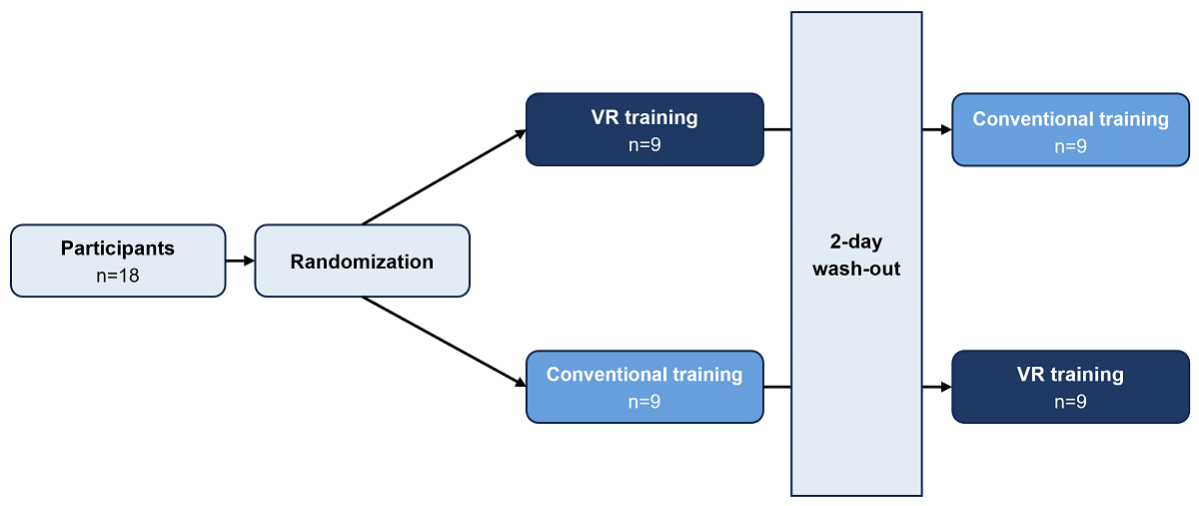
Study design. VR: virtual reality.

The participants were divided equally into 2 groups. Participants from each group received either 11 minutes of VR training or 11 minutes of conventional training and were switched to the other group after a 2-day wash-out period. Each session comprised a 3-minute warm-up, 5-minute main training session, and 3-minute cooldown session. The participants performed all the movements that were recorded on the video. Subsequently, the participants were evaluated for flow using a self-reported questionnaire. The study design is schematically illustrated in Figure 1.

### Ethics Approval

This study was approved by the Institutional Bioethics Committee of Chung-Ang University (1041078-201908-HRSB-231-01), and the experiment and research were conducted with written consent from all participants.

### Training Protocol

**Figure 2:**
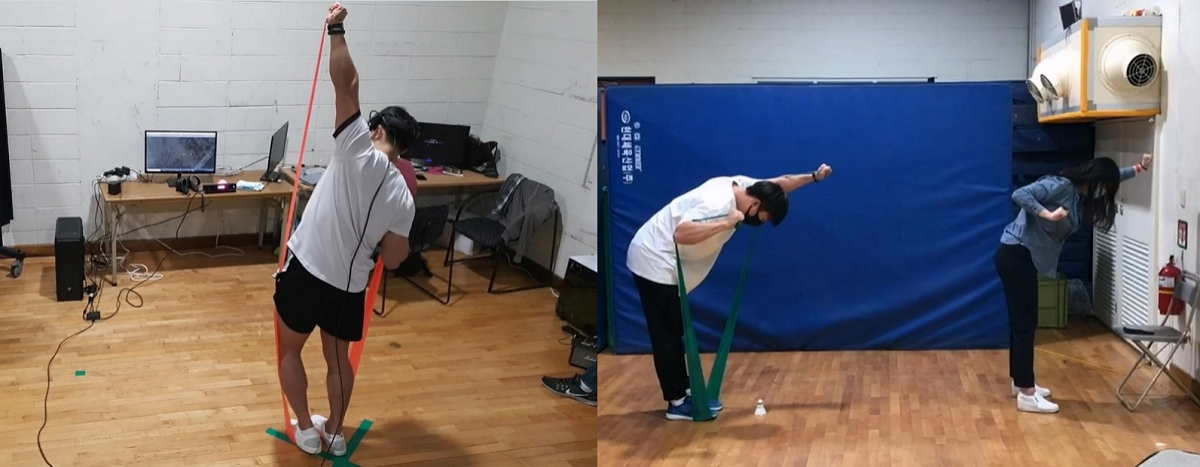
Virtual reality training and conventional training. Left: Participant receiving virtual reality training. Right: Participant receiving conventional training supervised by a female instructor.

At baseline, the participants were evaluated for the possible onset of motion sickness or epilepsy after wearing an HMD (Vive Cosmos Elite; High Tech Computer Corp). The training protocol consisted of 40 types of Pilates movements. These included 8 types of simple behaviors, 12 types of 2 complex behaviors, and 16 types of 3 complex behaviors. The participants in each group were instructed to perform 40 Pilates movements using a voice-recorded file, supervised by a female expert instructor (Figure 2).

Participants were allowed to interact with the VR environment using a Google Maps–based VR system (Vive Cosmos Elite). They were provided with views of tall mountains and the sky in a 3D video. They then felt as if they were flying through the valleys and sky while performing Pilates movements. Participants also performed Pilates movements under the guidance of a female expert instructor while listening to a voice recording.

### Study Procedure

Throughout this trial, the participants’ compliance and safety were meticulously monitored. At baseline, the participants were instructed to immediately quit Pilates movements as soon as they experienced nausea or dizziness while receiving VR-based training. They were instructed to wear the VR HMDs and a TheraBand (Hygenic Corp). However, in order to differentiate between the male participants and the female instructor, we used TheraBands with different colors. Subsequently, participants stood in a designated position. When participants sequentially performed all 40 Pilates movements in the VR environment, they received a response from the VR images.

### Measurements

#### Measurement of the Duration of Posture Maintenance

**Figure 3:**
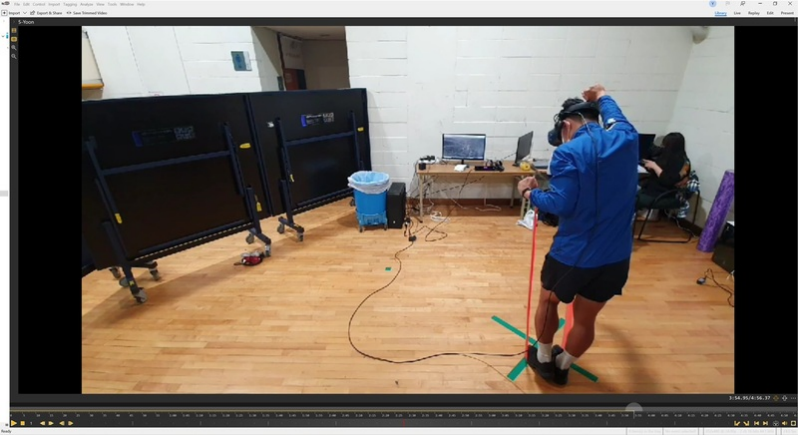
Measurement of the duration of posture maintenance through video analysis.

The duration of posture maintenance for the 40 Pilates movements was measured using sports video analysis (Figure 3). To simplify video recording, participants were instructed to perform the movements in a location where footprints were marked before starting the session. During the session, all movements were video-recorded using an Apple iPhone 11. Then, the duration of posture maintenance was measured (in seconds) using a sports video analysis program, Dartfish (Dartfish SA).

#### Measurement of Flow

After completing both sessions, participants filled out a flow questionnaire to identify the challenges, behaviors, goals, feedback, concentration, and control that they experienced while performing the Pilates movements. Measurements of reliability and validity have confirmed the acceptability of the flow scale as a single factor, thus indicating that it is useful across diverse samples of physical activity [21]. The applicability of the single factor of 10 items to various environments, such as volleyball, swimming, golf, dynamic sports, and static sports, is well documented in the literature [22-25].

### Data Analysis

Data were analyzed using GraphPad Prism (version 9.0; GraphPad Software) and SPSS Statistics (version 25.0; IBM Corp). First, a repeated-measures ANOVA (period × group) was performed to compare the duration of posture maintenance between the VR training and conventional training sessions. Second, a Pearson correlation analysis was performed to confirm the relationship between flow and the duration of posture maintenance. In all cases, the statistical significance level was set at *P*<.05.

## Results

### Verification of Difference in Duration of Posture Maintenance by Type According to Group and Time

**Figure 4:**
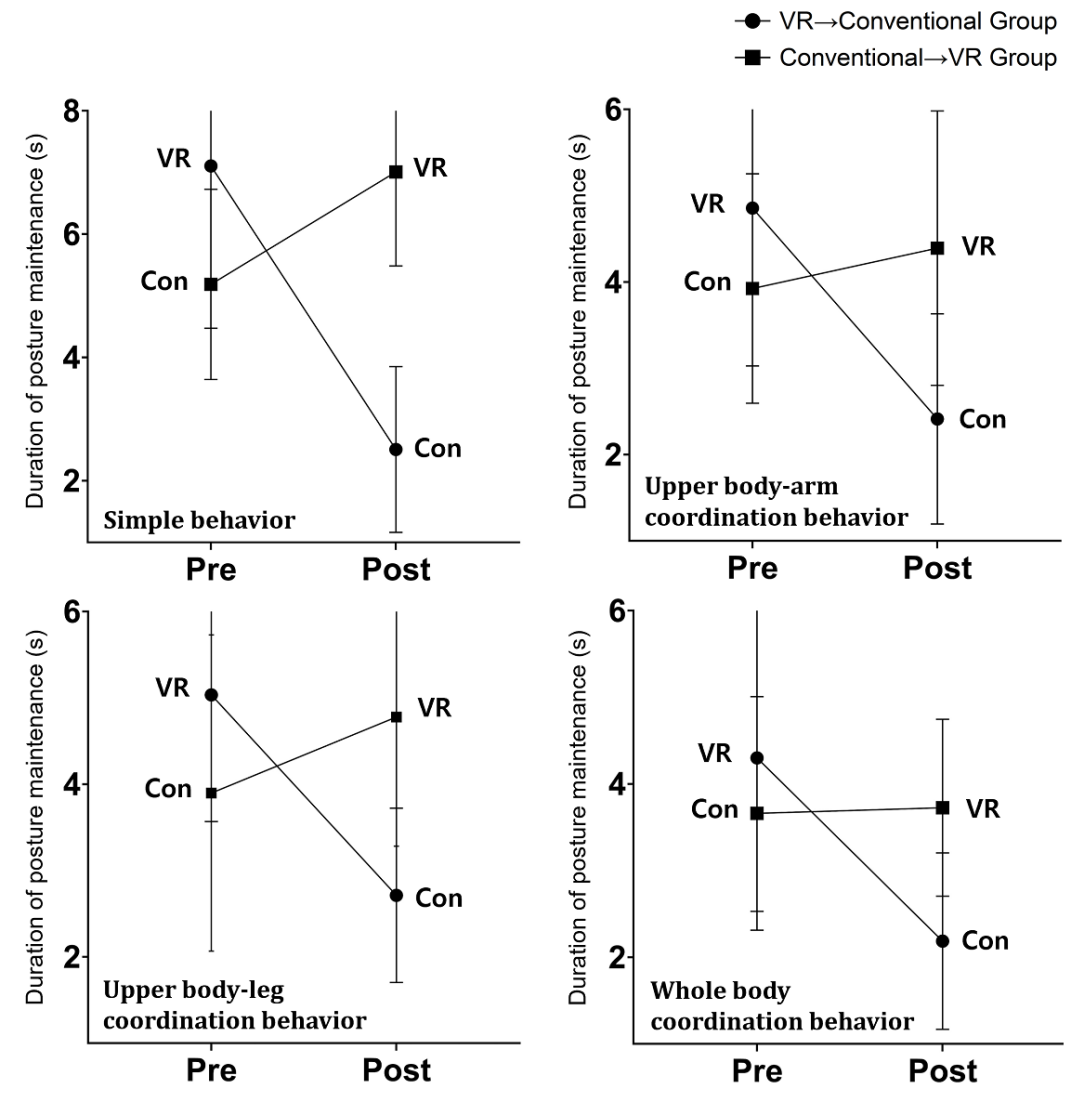
Interaction effects for all types of duration of posture maintenance among participants in a VR training session followed by a conventional session or vice versa. Con: conventional; VR: virtual reality.

Results for the duration of posture maintenance by type showed that simple behavior (*F*_1,16_=17.631; *P*<.001), upper body–arm coordination behavior (*F*_1,16_=6.083; *P*=.04), upper body–leg coordination behavior (*F*_1,16_=8.359; *P*<.001), and whole-body coordination behavior (*F*_1,16_=8.426; *P*<.001) all had an interaction effect. All groups had high posture maintenance in the VR sessions both pre- and postintervention compared to the conventional session; the group where the VR session was performed first had reduced posture maintenance during the conventional session, and the group where the conventional session was performed first had increased posture maintenance during the VR session (Figure 4).

### Verification of Difference in Flow by Type According to Group and Time

**Figure 5:**
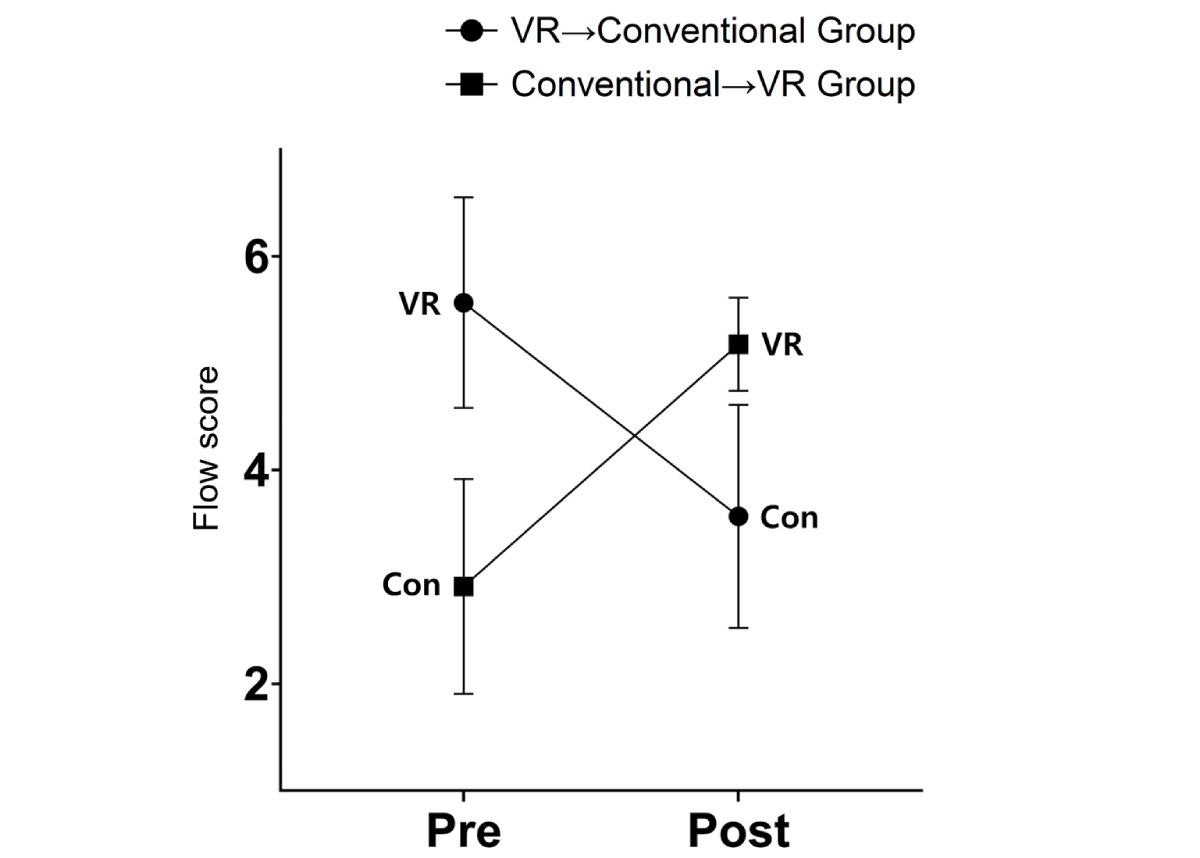
Interaction effects for flow among participants in a VR training session followed by a conventional session or vice versa. Con: conventional; VR: virtual reality.

Flow (*F*_1,16_=15.250; *P*<.001) showed an interaction effect. All groups had a higher degree of flow in the VR sessions, both pre- and postintervention, compared with conventional sessions. The group in which the VR session was performed first had reduced flow during the conventional session, whereas the group in which the conventional session was performed first had increased flow during the VR session (Figure 5).

### Correlation Between the Duration of Posture Maintenance and Flow

**Figure 6:**
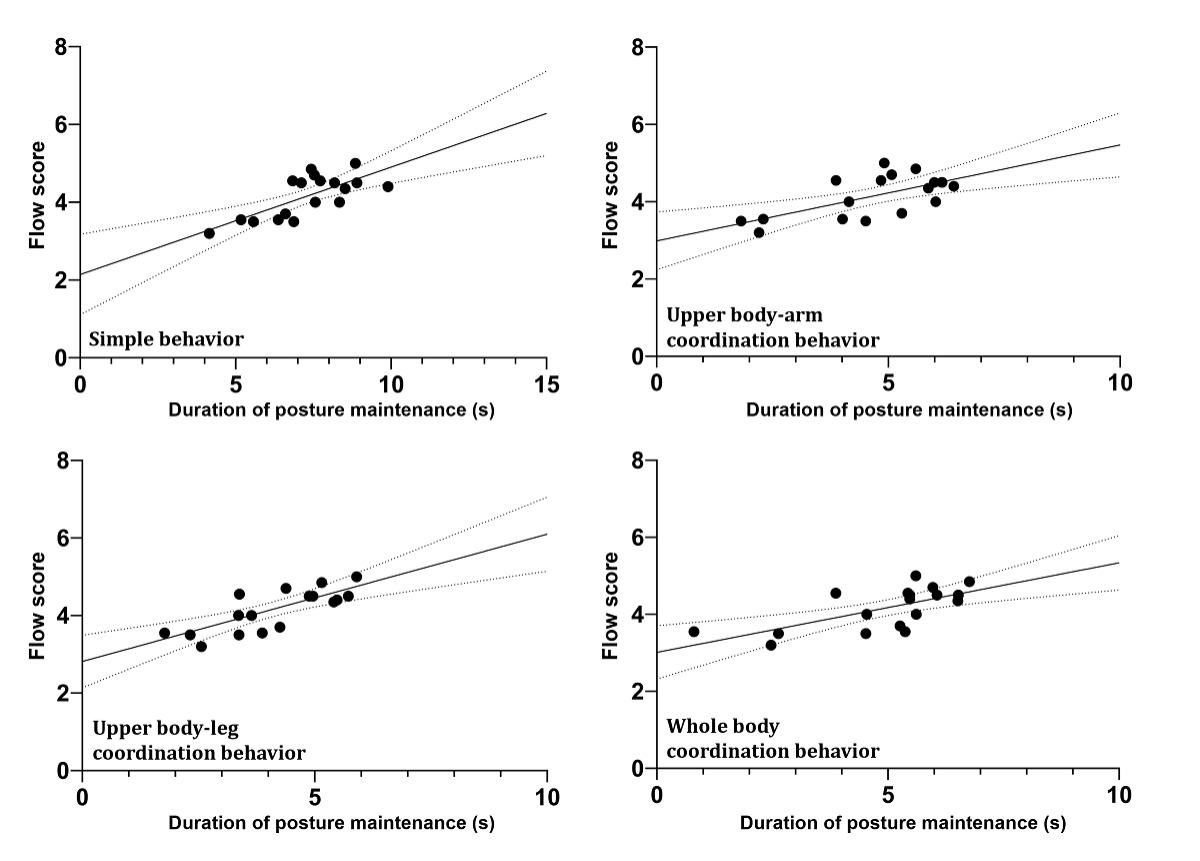
The results of Pearson correlation analysis for virtual reality training. There was a significant correlation between the duration of posture maintenance and flow in the virtual reality training group. Flow was positively correlated with simple behavior (r=0.49; *P*=.04), upper body–arm coordination behavior (r=0.56; *P*<.001), upper body–leg coordination behavior (r=0.70; *P*<.001), and whole-body coordination behavior (r=0.60; *P*<.001).

There were significant correlations between the duration of posture maintenance and flow in the VR training group. In detail, simple behavior was positively correlated with flow (r=0.49; *P*=.04), upper body–arm coordination behavior was positively correlated with flow (r=0.56; *P*<.001), upper body–leg coordination behavior was positively correlated with flow (r=0.70; *P*<.001), and whole-body coordination behavior was positively correlated with flow (r=0.60; *P*<.001). However, this was not observed in the conventional training group (Table 1 and Figure 6).

**Table 1:** Correlations between the duration of posture maintenance and flow (N=18).

Variable		*r*	*P* value
**Simple behavior and flow**			
	VR^a^ training	0.492	.04
	Conventional training	0.153	.32
**Upper body–arm coordination behavior and flow**			
	VR training	0.557	<.001
	Conventional training	0.342	.10
**Upper body–leg coordination behavior and flow**			
	VR training	0.702	<.001
	Conventional training	0.372	.09
**Whole-body coordination behavior and flow**			
	VR training	0.606	<.001
	Conventional training	0.436	.05

^a^VR: virtual reality.

## Discussion

### Principal Findings

Participants in each group received either VR training or conventional training followed by a 2-day washout period before switching to the other type of training. Each group performed 4 different types of Pilates movements in a VR environment and a conventional environment. After the session, participants then evaluated the flow of the exercise using a self-report questionnaire. In all filmed sessions, duration of posture maintenance was measured using a sports video analysis program. Repeated measures were used to compare duration of posture maintenance and flow between VR and conventional sessions. Pearson correlation analysis was then performed to determine the relationship between flow and duration of posture maintenance. The results show that training in a VR environment improves balance and postural stability, which in turn increases the amount of time a person can hold a particular position steady. Participants also experienced higher levels of flow in VR training and found that the VR environment increased their focus on the exercise and induced a higher level of flow state. Third, we found a correlation between flow and exercise duration during VR training, suggesting that the higher flow that occurs in the VR environment induces improvements in postural stability and holding time.

### Comparison to Prior Work

Movement duration was significantly different between the VR and conventional training sessions. A number of related studies have suggested 2 possible explanations for these results: improved posture and reduced resistance through VR. VR interventions have been shown to naturally promote exercise-specific behaviors through events presented in a virtual environment [26]. Furthermore, Lee and Kim [8] reported that sports VR training significantly influenced coordination related to balance and postural stability. In addition to this, various studies have found that VR simulation–based exercise increases body stability [27-29]. In other words, we can predict the mechanism by which VR increases action duration, as seen in this study: a sense of balance can enhance the stable continuation of a certain posture. On the other hand, Bryanton et al [30] reported that when children performed ankle flexion exercises in a VR environment for ankle joint rehabilitation, the duration of the dorsiflexion posture was enhanced compared to other environments, which may explain why the endurance required to fix and maintain a specific body pose for a long period of time is associated with training in the VR environment. Furthermore, previous studies showing that VR reduces the fatigue felt during isometric exercise may be related to the sustained effect of a similar mechanism in the static posture–holding movements performed in this study [31]. These findings suggest that VR environments may be useful in overcoming the negative sensations associated with exercise in a static position and modestly enhance performance potential. However, further studies that directly measure factors such as endurance and perceived resistance will be needed to prove this effect.

This study investigated whether VR environments provide an experience with high flow. After each session, participants reported that they felt more immersed in the VR training. Results from a randomized controlled trial evaluating a VR program for children with cerebral palsy highlighted maximizing engagement through flow in the VR environment as one of the strongest predictors of successful learning [32,33]. Vahle and Tomasik [34] reported that physical exercise in a VR environment led to a greater degree of flow as compared with a real-world environment, without respect to age. Moreover, according to Burt and Louw [35], VR technology was efficient in inducing a relatively higher level of flow. Virtual exercise environments can be characterized by a fundamental shift in focus from exercise to flow activity participation. While participants may perform physical movements of their own volition, the phenomenon of performing naturally guided behaviors in response to the interface provided by VR can also be explained. In this study, participants experienced flow states while performing the exercises, so it can be assumed that they adapted to the difficulty of the challenge and felt a sense of mastery. This is supported by research that suggests that flow is related to the balance between an individual’s athletic and psychological abilities [36].

We conducted a correlation analysis to determine whether the participants adapted to the difficulty of the challenge and felt a sense of mastery over the duration of posture maintenance during training in the VR environment. The analysis confirmed a correlation between duration of posture maintenance and flow during VR training sessions. Virtual environments have been shown to induce psychological changes that engage participants in exercise [37] and enhance motor learning, including balance and posture [38]. These findings can be interpreted to suggest that performing physical activities while watching videos provided by VR devices increases motor focus. Specifically, participants experienced the possibilities and benefits of flow during physical activity combining Pilates and exergames in a virtual environment while performing naturally guided behaviors to respond to the videos provided in the game. VR exercise environments add virtual elements to real-world sensory elements and graphics to provide new challenges during real-world exercise [39,40]. Therefore, the findings of this study support the notion that VR training can provide seamless control over movement, as it allows for a gradual progression of the exercise protocol while maintaining the participant’s attention. Furthermore, Mouatt et al [41] reported that VR-based flow engagement can have dramatic effects, especially for low-intensity exercise, by providing a virtual exercise environment for both able-bodied and disabled participants. In another study, an 8-week VR exergame training program designed to improve physical conditioning and increase performance yielded similar results to this study [42]. Virtual environments have also been proven to provide more intense visual stimulation than conventional physical activity, inducing changes in brain mechanisms responsible for high levels of concentration, thereby improving motor learning and body control, which are necessary for balance [43]. In another study, the authors interviewed users of a VR exercise program to gain insight into their experiences [44]. The participants expressed feelings of being in the environment as if they were floating in the air, able to observe the landscape in 360 degrees, and a sense of accomplishment after achieving what they wanted through postural control. The results of this study and various previous studies suggest that participants’ flow responses are associated with increased playtime and improved performance in the long run. The psychological mechanisms of flow can be applied to dynamic and challenging exercises, physical activities, and even static, near-isometric movements in VR environments.

### Strengths and Limitations

The VR environment’s ability to improve an individual’s stability in maintaining posture can be considered a major contribution to Pilates and physical activity. Traditionally, research avenues exploring physical activity and sports within VR realms have predominantly focused on the effects and efficiencies concerning dynamic performance parameters, such as strength enhancement and aerobic capacity. Nonetheless, this study ventured beyond the conventional boundaries by elucidating the efficacy of VR training methodologies specifically tailored for low-intensity, static movements and activities, wherein the core focus is balance retention and maintenance. Thus, this study not only corroborated the increasing importance of VR’s flow environment but also exemplified its potential to enhance the quality of physical activities through improved postural stability and maintenance mechanisms.

There are several limitations to this study. First, it involved a small number of participants, which makes it difficult to generalize to the entire population. Therefore, a larger study that randomizes men and women in equal proportions and includes participants of different ages is necessary to generalize the findings. Second, this study focused on demonstrating the effect on duration of posture maintenance of a VR environment in relation to flow. Future research should expand the factors measured and types of physical activity to demonstrate the strengths of VR in improving postural stability and increasing duration from a variety of perspectives. Third, this study evaluated the effectiveness of VR training in the short term, so further research is needed to identify any adverse effects and obtain new insights that may have been difficult to detect in a short-term intervention when applied in the long term.

### Conclusion

In conclusion, the findings of this study cautiously suggest that VR Pilates training might be more effective in improving duration of posture maintenance and inducing higher flow than conventional Pilates training. However, careful judgment is needed when asserting that physical activities in a VR environment are absolutely more beneficial than conventional training. Nonetheless, it suggests the possibility that a VR environment can enable effective performance even with different types of movement and when the difficulty of the applied movements increases in physical activity. In the future, there remains the challenging task of minimizing chronic stressors such as motion sickness and the weight of the device. Moreover, there is a need to develop distinct physical activities and exercise programs to overcome these challenges. We cautiously predict that if such efforts persist, useful VR environment training could be realized.

## Appendix

Multimedia Appendix 1 CONSORT (Consolidated Standards of Reporting Trials) 2010 checklist.

## Abbreviations

HMD: head-mounted display
VR: virtual reality

## Data Availability

The data sets generated and analyzed during this study are not publicly available for privacy and ethical reasons but are available from the corresponding author upon reasonable request.
